# Effects of exposure to large sharks on the abundance and behavior of mobile prey fishes along a temperate coastal gradient

**DOI:** 10.1371/journal.pone.0230308

**Published:** 2020-03-16

**Authors:** Brendan D. Shea, Connor W. Benson, Christine de Silva, Don Donovan, Joe Romeiro, Mark E. Bond, Scott Creel, Austin J. Gallagher

**Affiliations:** 1 Beneath the Waves, Herndon, Virginia, United States of America; 2 Three Seas Program, Northeastern University, Nahant, Massachusetts, United States of America; 3 Thayer Academy, Braintree, Massachusetts, United States of America; 4 333 Studios, Exeter, Rhode Island, United States of America; 5 Florida International University, North Miami, Florida, United States of America; 6 Department of Ecology, Montana State University, Bozeman, Montana, United States of America; 7 Department of Marine and Environmental Sciences, Marine Science Center, Northeastern University, Nahant, Massachusetts, United States of America; Institut de recherche pour le developpement, FRANCE

## Abstract

Top predators can exert strong influences on community structure and function, both via direct, consumptive effects, as well as through non-consumptive, fear-based effects (i.e. predation risk). However, these effects are challenging to quantify, particularly for mobile predators in marine ecosystems. To advance this field of research, here we used baited remote underwater video stations (BRUVs) to assess how the behavior of mobile fish species off Cape Cod, Massachusetts, was affected by exposure to large sharks. We categorized sites into three levels of differential shark predation exposure (white sharks, *Carcharodon carcharias*) and quantified the relative abundance and arrival times (elapsed time before appearing on screen) for six mobile fish prey groups to the BRUV stations. Increased large shark exposure was associated with a decrease in overall prey abundance, but the overall response was prey group-specific. Foraging of smooth dogfish, a likely important prey item for large sharks in the system, was significantly reduced in areas frequented by white sharks. Specifically, the predicted probabilities of smooth dogfish bait contacts or bite attempts occurring were reduced by factors of 5.7 and 8.4, respectively, in areas of high exposure as compared to low exposure. These modifications were underscored by a decrease in smooth dogfish abundance in areas of high exposure as well. Our results suggest that populations of large, roving sharks may induce food-related costs in prey. We discuss the implications of this work within the context of the control of risk (COR) hypothesis, for the purposes of advancing our understanding of the ecological role and effects of large sharks on coastal marine ecosystems.

## Introduction

Top predators can exert strong influences on community structure and function, both via direct, consumptive effects (i.e., killing/predation) as well as through non-consumptive, fear-based effects (risk effects)[1]. The importance of risk-based effects of predators is well-established[2,3] and they have the potential to trigger trophic cascades[4–7]. Controlled field experiments have demonstrated that behavioral responses of prey species under exposure to predators can affect foraging, habitat selection and reproductive fitness[8,9], and play an important role in overall community structure[10].

Applying these lessons and concepts to ecological studies of free-ranging large predators and prey (e.g., large mammals and fishes) remains challenging, due to the difficulties in monitoring large animals and the high rates of movement of top predators[11]. The risk of predation experienced by prey species varies throughout each habitat both spatially and temporally[12–16], and behavioral responses to this variation in risk, such as increased vigilance, can be difficult to measure[17,18]. Yet, work is beginning to show that large predators can have profound impacts on prey abundance, behavior, and physiology[19–22], and better evaluating these patterns may help improve our understanding of the ecological role of top predators[23,24] as well as the management challenges that face them[25]. Sharks are among the most well-known, and threatened, predators in the marine environment[26,27], yet the mechanisms by which they may influence their prey are not fully understood and thus remain debated[28–30]. Recent studies have suggested that context is important when evaluating the effects of mobile sharks on prey, as the strength of predator effects can vary due to numerous variables, including predator hunting behavior and/or functional attributes, environmental conditions, landscape features, and the predictability of risk itself [11,21,31–33].

Responses that reduce risk are affected by their costs, which are mediated in some cases by foraging modifications and in some cases by physiological stress responses. The control of risk (COR) hypothesis suggests that if predation risk is predictable and controllable, antipredator responses will be associated with food/safety trade-offs, but if predation risk is unpredictable or uncontrollable, it will be associated with physiological stress responses[34]. Indeed, in a study in the Greater Yellowstone Ecosystem, elk outfitted with GPS collars were found to avoid preferred grassland grazing areas when wolves were nearby, due to the elevated risk of predation in open spaces[35]. Conversely, elevated and unpredictable predation risk has been observed to cause chronic stress in snowshoe hares[36].

White sharks (*Carcharodon carcharias*) are the largest predatory shark in the world and are renowned for their occurrence at numerous ‘hot-spots’ globally that serve as natural foraging areas. In these areas, white sharks seasonally hunt seals and other large pinnipeds, and studies have shown this temporal spike in predation risk can elicit both behavioral and physiological responses in their prey[21,37]. As predicted by the COR hypothesis, seals exposed to unpredictable and uncontrollable white shark predation had increased glucocorticoid stress hormone levels relative to seals at places and times with little risk of shark predation. Also as predicted, stress responses were stronger for seals at sites with little structural cover, which seals use to control their exposure to predation by sharks[21,37].

The coastal waters of Cape Cod, Massachusetts comprise a white shark ‘hot-spot’, where subadult and adult sharks congregate to feed on gray seals (*Halichoerus grypus*) from July to October[38,39]. However, white sharks are known to also consume a variety of teleost and elasmobranch fishes throughout their ontogeny[40,41] and range in the Northwest Atlantic[42]. In this region, there are numerous fish species that serve as additional prey for white sharks, including smooth dogfish (*Mustelus canis)*, bluefish (*Pomatomus saltatrix*), northern and striped sea robins (*Prionotus carolinus*/*Prionotus evolans*), and winter and little skates (*Leucoraja ocellate*/*Leucoraja erinacea*)[43,44]. Whereas the movements and connectivity of white sharks off the eastern coast of the United States have been studied[39], the mechanisms by which sharks affect their prey through physiological and behavioral modifications are not yet well known. A recent study evaluating the physiological effects of exposure to white sharks on a local fish species, the striped bass (*Morone saxatilis*), found no differences in baseline levels of blood stress indicators, again supporting the COR hypothesis and suggesting that for some species, the non-consumptive effects experienced may be behavioral rather than physiological[45].

In recent years, an increasing number of studies have used baited remote underwater video stations (BRUVs) in order to non-invasively sample mobile fish populations, a technique that can detect elusive species which may be missed by underwater visual census[46] and is particularly effective for mobile fishes and for sampling in areas where fish may be scarce[47]. Several studies have used BRUVs to analyze predator-prey dynamics involving sharks[15], and to examine how predation risk influences behavior in mobile fish species[33,48,49]. The majority of BRUV studies have been performed on hard substrata on and around coral and rocky reefs, both due to the expectation for increased biodiversity on videos collected near reefs, as well as the improved visibility resulting from a lack of loose sediments[50]; however, BRUVs have also been utilized to study fish assemblages in unconsolidated, high sediment marine environments like those in the temperate waters of the Northwestern Atlantic[51].

Here, we used BRUVs to investigate whether increased potential encounter rates with large sharks induced effects on the behavior of mobile fishes in the coastal waters surrounding Cape Cod, Massachusetts. Our objective was to record and analyze the occurrence and behavior of fish communities living in areas of differential shark exposure. BRUVs were deployed over a 4-month period in order to evaluate behavioral responses in potential prey items (i.e., mobile fishes) in otherwise similar (habitat complexity, temperature, depth) locations across a gradient of shark occurrence and potential exposure, a proxy for predation risk. We hypothesized that due to prey aiming to minimize encounters with potential predators, prey species would be less abundant and slower to arrive at the BRUV stations in areas more commonly frequented by sharks. Furthermore, we hypothesized that foraging behaviors would be suppressed in areas of higher exposure, due to a need for increased vigilance. We discuss the implications of this work on evaluating the ecological role and effects of large predators on coastal marine ecosystems.

## Methods

### Study area

This study was conducted from June 7, 2018 through September 27, 2018 off the lower and outer coasts of Cape Cod, to capture the seasonal abundance of large marine predators in the region [39]. Since a primary objective of this study was to evaluate landscape-level effects of predation risk on prey, we determined sampling locations by estimating the potential exposure rates (from high to low) of white sharks on habitats along the coast, using a two-pronged approach. First, we used a publicly available acoustic open-sourced, mobile application for reporting and displaying regional white shark sightings and detections, based on public sightings and acoustic telemetry data (Sharktivity, Atlantic White Shark Conservancy)[45]. Based on the available data from 2016–2018, we chose four study locations along a north-south gradient on the outer coast of Cape Cod and designated them to be “high exposure”, as white shark sightings were consistently clustered in these areas, year after year (Sharktivity, Atlantic White shark Conservancy). We also selected two “low exposure” sites, to capture data on fish abundance and behavior in the absence of white sharks–one site inside Pleasant Bay, in Chatham, MA and another in the waters of Nantucket Sound, to the west of Monomoy Island. There were only two reports of white sharks within Pleasant Bay available on Sharktivity, both of which occurred near the harbor entrance, and no shark sightings were seen or reported in Nantucket Sound, where white sharks rarely occur (G. Skomal, pers. communication).

**Fig 1:**
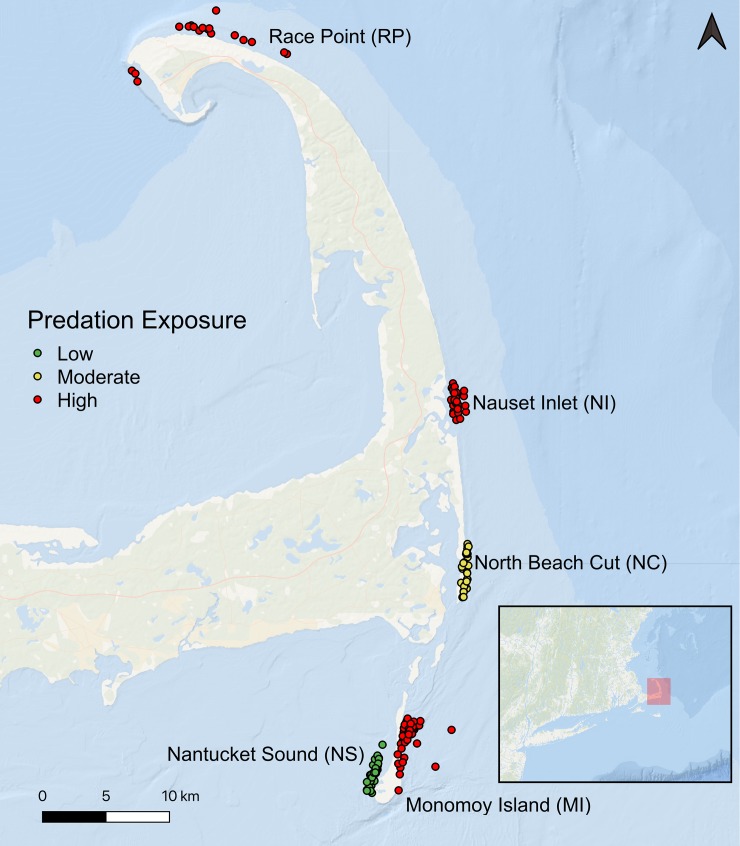
Map of study sites and depiction of BRUV deployments. Map of BRUV deployments off the outer and lower coasts of Cape Cod, Massachusetts, USA. Exposure levels at each site are indicated by the color of the markers.

We cross-checked our qualitative exposure categorizations with empirical observations of white shark occurrence (occupancy on camera) across these sampling locations during the present study, only in instances when there were opportunistically detected by our cameras (see methods below). This exercise suggested that at least one site (North Beach Cut) previously considered ‘high exposure’ showed significantly lower occupancy of transient white sharks than the others in this category, leading us to designate this spot as “moderate exposure”. Additionally, footage collected in Pleasant Bay was ultimately excluded from analysis due to very poor visibility resulting in a lack of data. The final study design thus comprised five sites which were characterized (from South to North) as follows: Nantucket Sound (NS, low exposure); Monomoy Island ocean side (MI, high exposure); outside the North Beach Cut in Chatham, MA (NC, moderate exposure); Nauset Inlet (NI, high exposure); and Race Point (RP, high exposure; Fig 1).

We deployed BRUVs on sandy bottom at various depths ranging from 2 to 13 meters (mean 5.77 ± 1.96 meters). All areas exhibited consistent environmental conditions, depth, and substrate, with localized areas of rock/cobble substrate. None of the sites were characterized by algal canopies or beds. Deployments were randomly conducted across all tide heights and states, avoiding areas of heavy currents which could tip the BRUV during sampling or lead to confounding environmental differences.

### BRUV design and deployment

**Fig 2:**
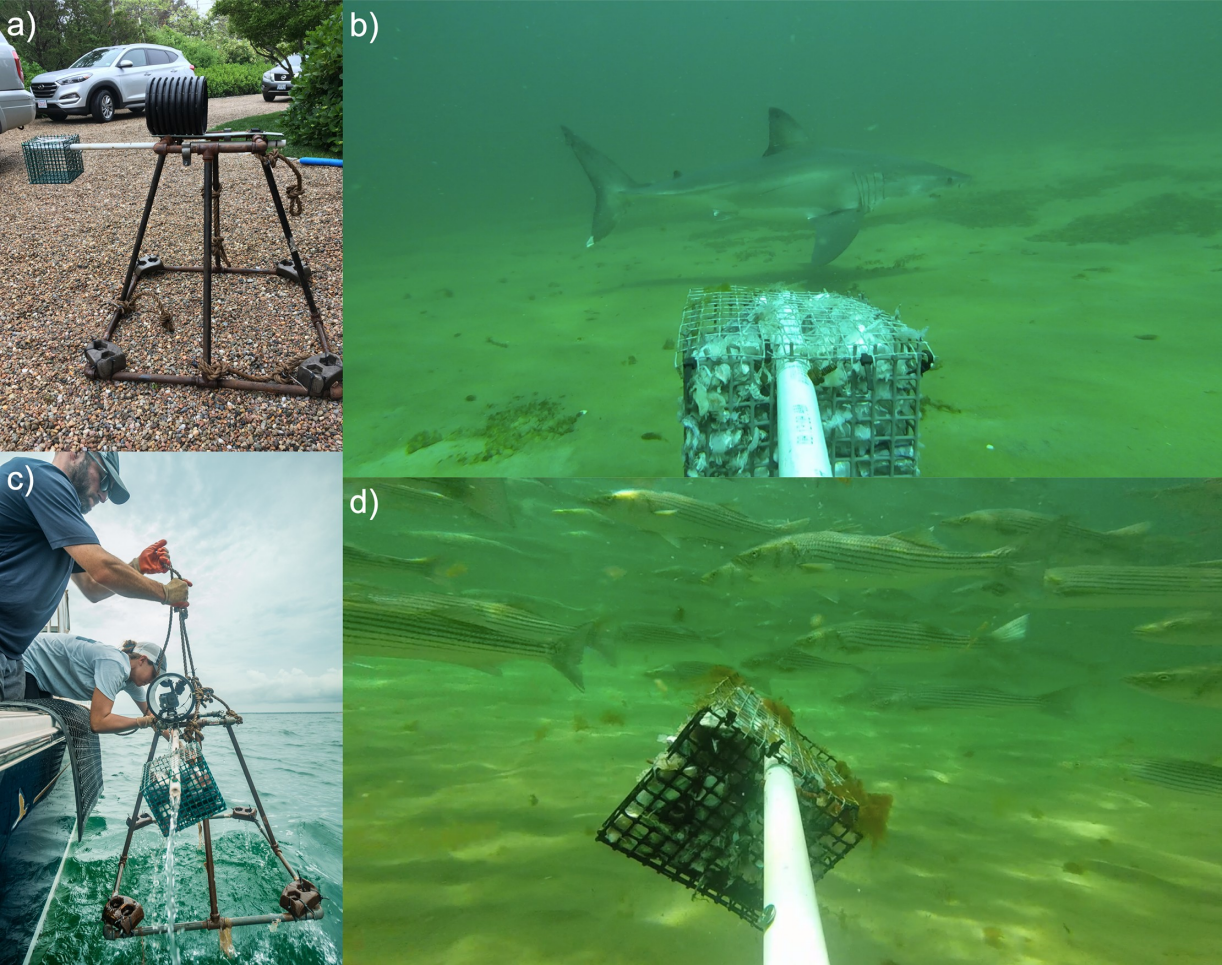
BRUV design and image captures. (a) BRUV assembly on dry land; (b) Still image from BRUV deployment with an opportunistically recorded white shark (*Carchardon carcharias*) in frame; (c) BRUV assembly, attached to marker buoy and with camera in place. Photo taken during BRUV retrieval; (d) Still image from BRUV deployment with a school of striped bass (*Morone saxatilis*) in frame.

We used BRUV stations to make observations of mobile fish communities at our study sites[52]. Each BRUV station consisted of a 64 cm-tall, trapezoidal-prism frame constructed using steel pipes (Fig 2). A steel block weighing approximately 3 kg was affixed to each of the bottom corners of the frame to stabilize the BRUVs, resulting in a total weight of approximately 22 kg for each frame. The bait crates consisted of approximately 15x15 cm cube-shaped chum pots constructed from 16-gauge PVC-coated marine wire forming a 2.13 cm mesh. Bait crates were mounted to the BRUV frame using bait arms comprising 2.13 cm-diameter PVC pipe, which were attached to extend laterally from the top of the BRUV such that the bait crate was positioned approximately 30 cm from the frame. Camera housings were constructed using 16.88 cm-diameter perforated PVC pipe, and then mounted atop the BRUV to protect the cameras in the event of tipping.

To record fish behaviors, we utilized high-definition action cameras (GoPro Hero and GoPro Hero+), which were secured within the protective housing and pointed to face outward, looking down the bait arm to the bait crate with an estimated 160° field of view. Cameras were programmed to shoot at 60 frames per second at either 720p (GoPro Hero) or 1080p (GoPro Hero+) resolution, and mounted at a slightly downward angle, approximately 10° below horizontal, in order to record the bait crate as well as the area beyond. Prior to each deployment, bait crates were filled with approximately 1,250 grams of chopped mackerel. We deployed BRUVs from a research vessel by lowering them to the sea floor using a rope attached to a surface marker buoy, and then retrieved them after collecting one hour of undisturbed footage. A 5-minute delay was incorporated into the start of each sampling period to allow conditions to return to ambient following deployment of the BRUV and disturbance of the seafloor. Due to camera battery life restricting the video collection, for a small number of replicates (n = 8), the 5-minute delay was slightly shortened (<30 seconds) such that a full hour of footage could be analyzed. Water depth and temperature were also recorded at each sampling location. Simultaneous replicates were placed 250 meters or more from one another. After deployment, the boat left the sampling area to avoid disturbance, until the sampling window was up and we returned to collect our equipment.

### Video analysis

Videos were visually analyzed to identify trends in prey behavior and abundance. In order to evaluate the influence of shark exposure, we assessed species assemblages and quantified several prey behaviors. For each replicate (i.e., one BRUV drop with one hour of footage), we quantified the relative abundance (MaxN, the maximum number of individuals on screen at once[50]) of white sharks and of each prey group: smooth dogfish; skates (winter and little skate); striped bass; bluefish; flounder (winter flounder and summer flounder–*Pseudopleuronectes americanus*/*Paralichthys dentatus*), and sea robins (northern and striped sea robins). MaxN is a widely-used metric in BRUV studies which provides a conservative estimate of relative abundance that can be used to compare differences in fish [50]. To compare behaviors, we both quantified arrival time for each prey group and recorded specific foraging behaviors. Arrival time, referred to in other BRUV studies as “T1st”, represents the time elapsed (in seconds) prior to the first appearance on screen by an individual of a given prey group[50]. We quantified foraging behaviors at the prey group, rather than the individual, level, due to the challenges associated with identifying specific individuals. For each prey group, we recorded: bait contacts (binary; yes or no); the number of actual bites at the bait crate (which were then normalized; see Statistical Analysis); and bait residency. Bait residency was measured in seconds, where the first individual from each prey group to make contact with the bait crate was monitored and timed until it was approximately 3 body lengths from the bait[33]. Bait residency was only calculated for the first individual from each prey group.

Whereas our primary objective was to record mobile fish communities, we were able to opportunistically record a small number of white shark occurrences (n = 31), and we used this information to validate the exposure classification of the study locations. We recorded white shark occupancy, defined as the total time elapsed with a white shark in frame for each video, as a supportive proxy for shark exposure.

### Statistical analysis

In order to determine which effects should be included as independent variables for analysis, we explored potential trends in response variables associated with environmental conditions such as temperature and depth via univariate models. We compared environmental differences among study sites using a series of One-Way Analyses of Variance (ANOVAs). To validate the relative shark exposure levels used to classify our study sites, we visualized trends and compared mean white shark occupancy across sites using a series of Welch’s T-Tests. A negative binomial generalized linear model (GLM, family = negative binomial; link function = log) was used to analyze relative prey abundance with respect to shark exposure and temperature. We also ran a negative binomial GLM on prey arrival times, using shark exposure and temperature as the independent variables. Non-arrivals (i.e., videos where a prey family did not appear on the camera) were excluded from analysis. A series of Likelihood Ratio Tests (LRTs) was applied to each GLM to determine the significance and relative strength of each model component.

Smooth dogfish were the prey group that most frequently interacted with the bait, providing an opportunity to evaluate prey-specific foraging behaviors. We isolated replicates where smooth dogfish occurred, and first used a quasibinomial GLM (family = quasibinomial; link function = logit) to assess the probability of smooth dogfish contacting the bait, either with an investigatory bump or a bite attempt. We then compared bait residency times among exposure levels using a negative binomial GLM, excluding videos where no bait contact was made. We analyzed the likelihood of smooth dogfish biting the bait using the same methods as bait residency. For replicates where bites occurred, we normalized the total number of bait bites by the corresponding MaxN value to represent the relative bait interest by any one individual. We compared these normalized bite rates using a negative binomial GLM, with shark exposure and temperature as independent variables. In order to determine if any overall differences in dogfish foraging would be biased by acute predator avoidance behaviors in instances where white sharks actually occurred, we isolated replicates from sites where white sharks occurred at any point during the study (moderate and high exposure), and compared smooth dogfish foraging behaviors via quasibinomial GLMs, using white shark occurrence (binary; yes or no) and temperature as predictors. For a complete description of analyses and predictors, please see S1 Table.

### Ethics

No animals were captured, touched, or manipulated in any way. No animals were provisioned or consumed the bait. All experiments were conducted in accordance with the standards set by the Canadian Council of Animal Care (CCAC). Due to the observational nature of the study, no formal animal care or ethics approvals or permits were needed. The individuals pictured in this manuscript have given written informed consent (as outlined in PLOS consent form) to publish these case details.

## Results

### Video collection and analysis

We completed 220 BRUV deployments in total, of which 49 (22%) were excluded due to tipping of the station, camera failure, or drift algae significantly obstructing the camera’s view, resulting in 171 hours of usable video. Additionally, all replicates from one of the low exposure sites (n = 26), Pleasant Bay, was discarded due to very poor visibility and a resulting lack of data, leaving 145 hours of footage from five sites for the subsequent analysis. Across all useable BRUVs we observed at least 11 teleost species, 6 elasmobranch species, 8 crustacean species, gray seals, diving cormorants, and an unidentified species of squid. Exploratory analyses showed no significant differences in depth among sites (see S1 Fig), so depth was excluded from any future modeling. Temperature, however, varied significantly among sites and by month, and was thus retained as an independent variable in all model runs.

### White shark occupancy

**Fig 3:**
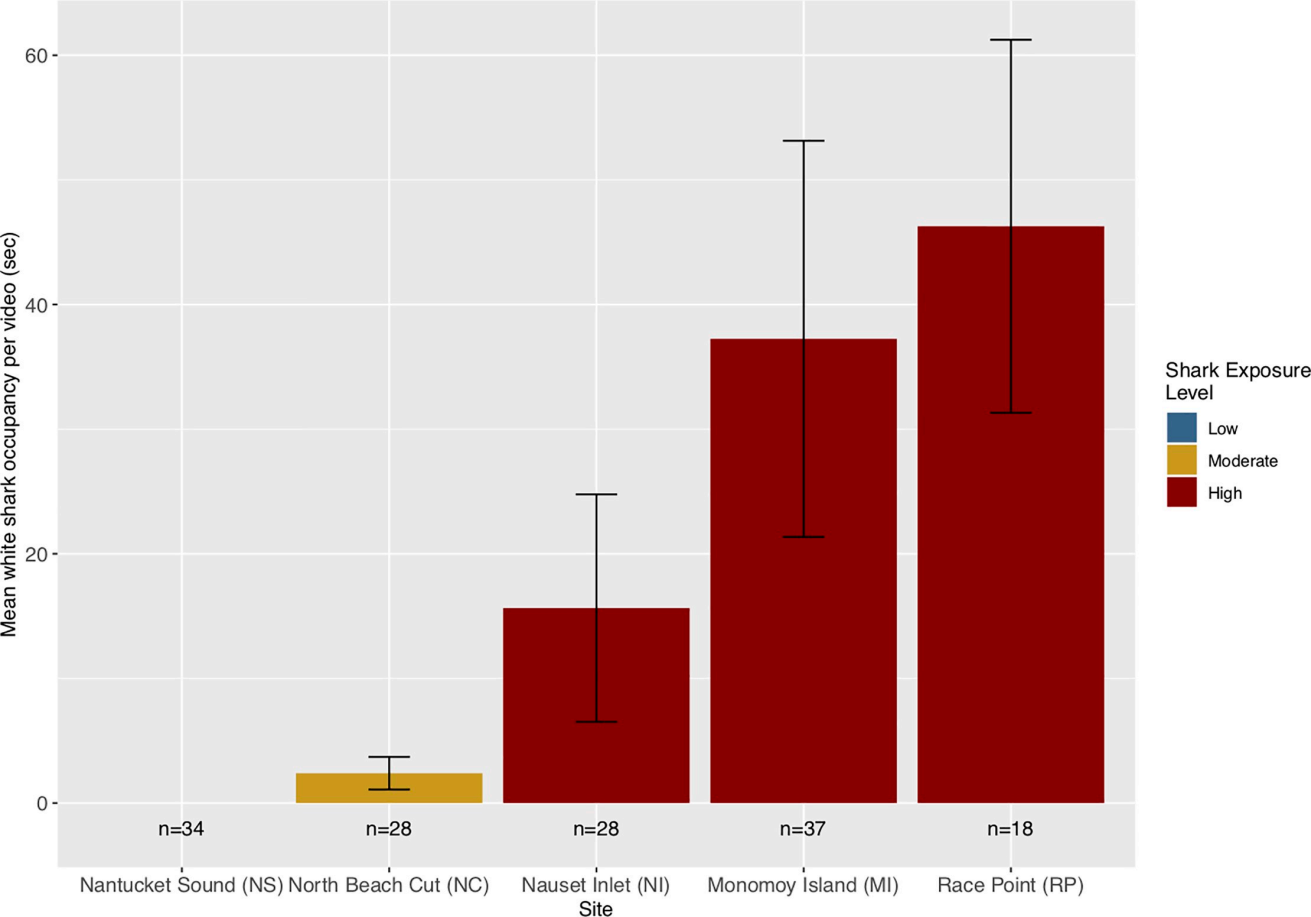
Shark occupancy. Normalized mean occupancy (seconds +/- SE) of white sharks in frame during 60-minute recordings at each site. Colored bars indicate shark exposure levels used for statistical analysis. The total number of deployments at each site is indicated beneath each bar.

To cross-check our *a priori* categorization of differential shark exposure levels, we visualized the occupancy of white sharks on videos collected from each site by recording the total time with a white shark in frame for each video, normalized using the total number of deployments at each site (Fig 3). No sharks were ever recorded on video at the low exposure site. Normalized white shark occupancy at the NC site (moderate exposure) was 2.4 seconds per hour (sec/hr), significantly lower than the mean normalized occupancy at high exposure (RP, MI, and NI; mean = 31.9 sec/hr) sites (Welch’s T-Test; t = -3.47, df = 85.87, p<0.001). There were no differences in white shark occupancy among high exposure sites (Welch’s T-Tests; NI and MI: t = 1.18, df = 55.60, p = 0.24; NI and RP: t = -1.75, df = 29.43, p = 0.09; RP and MI: t = -0.41, df = 48.09, p = 0.68).

### Prey abundance

**Fig 4:**
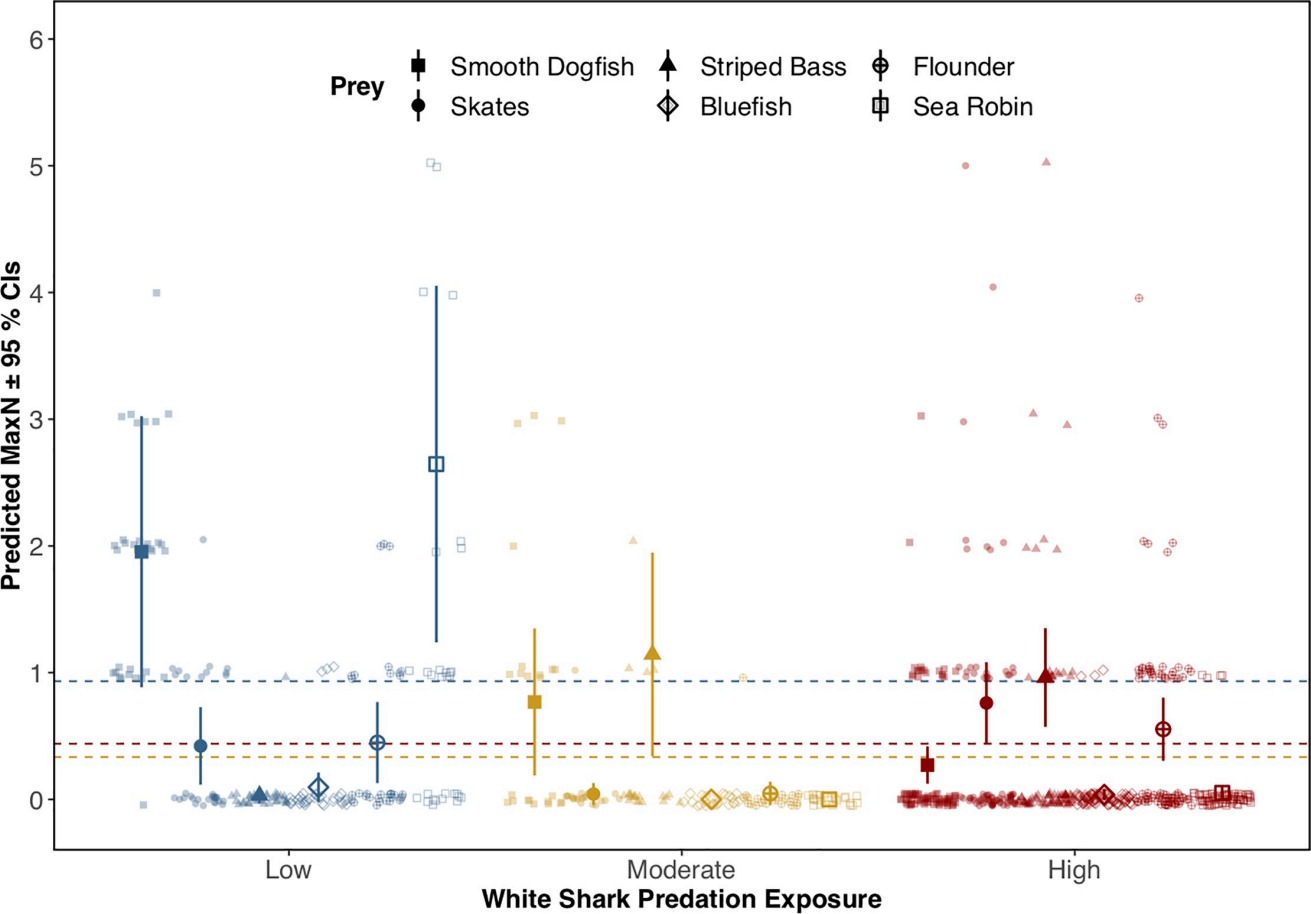
Predicted prey abundance across a gradient of shark exposure. Predicted prey abundance (MaxN; ± 95% confidence intervals) from a negative binomial Generalized Linear Model (GLM) for six different prey groups across varying levels of shark exposure. Predicted values, as well as mean abundance by exposure level (dashed lines), are overlaid on top of raw observational data. Confidence intervals are not included for prey groups not observed within a given exposure level.

Our model produced significant effects on prey abundance (MaxN) that corresponded with the interaction between the level of shark exposure and prey group (negative binomial GLM, LRT p<0.001, Table 1). Both shark exposure and temperature were strongly associated with variations in overall prey abundance (LRTs p<0.001), though LRTs applied to the model indicated that the interaction between exposure and prey group corresponded with the strongest overall effect. Overall prey abundance was greatest at the low exposure site and was positively associated with warmer water temperatures (p<0.001). Smooth dogfish were most abundant at the low exposure site (negative binomial GLM; predicted MaxN = 1.95 ± 1.35; p<0.001; Fig 4) and least abundant at the high exposure site (predicted MaxN = 0.027 ± 0.01, p<0.01). Sea robins were also least abundant at the high exposure site (predicted MaxN = 0.05 ± 0.002; p<0.001). For three of the six prey groups, however, predicted abundance was greatest at the high exposure sites: skates (predicted MaxN = 0.76 ± 0.03; p<0.01); striped bass (0.96 ± 0.04, p<0.001); and flounder (0.55 ± 0.03; p<0.05). Predicted total abundance, however, was significantly greater at low exposure sites (predicted MaxN = 0.93 ± 0.08) than at high exposure sites (predicted MaxN = 0.43 ± 0.02; p<0.001), suggesting that this overall trend was driven largely by smooth dogfish.

**Table 1:** Effects of shark exposure on relative abundance of prey groups.

	Coefficients	Estimate	Standard Error	Z-value	Pr(>|z|)
	Intercept (Low + Smooth Dogfish)	-2.484	0.685	-3.63	<0.001*
					
**Low Exposure**	Skates	-1.533	0.451	-3.40	<0.001*
	Striped Bass	-4.151	1.057	-3.93	<0.001*
	Bluefish	-3.018	0.671	-4.50	<0.001*
	Flounder	-1.473	0.446	-3.30	<0.001*
	Sea Robins	0.306	0.373	0.82	0.413
**Moderate Exposure**	Skates	-2.912	1.129	-2.58	<0.01*
	Striped Bass	0.398	0.514	0.77	0.439
	Bluefish†	-29.90	448900	0	0.999
	Flounder	-2.809	1.083	-2.59	<0.01*
	Sea Robins†	-29.90	448900	0	0.999
**High Exposure**	Skates	1.034	0.333	3.11	<0.01*
	Striped Bass	1.265	0.327	3.86	<0.001*
	Bluefish	-1.988	0.659	-3.02	<0.01*
	Flounder	0.716	0.342	2.09	<0.05*
	Sea Robins	-1.672	0.585	-2.86	<0.01*
	Temperature	0.163	0.030	4.94	<0.001*
**Likelihood Ratio Tests (LRTs)**	**Effect**	**Likelihood Ratio ChiSq**		**Degrees of Freedom**	**Pr(>ChiSq)**
	Exposure	9.386956		2	9.15E-03*
	Temperature	20.103		1	7.34E-06*
	Exposure:Prey	234.14		15	2.26E-41*

Summary results of a negative binomial GLM assessing the effect of shark exposure and temperature on the abundance (MaxN) of six prey groups, and summary of LRTs.

* Indicates significance at the α = 0.05 level.

† Prey group not observed at moderate exposure site.

### Behavior

**Fig 5:**
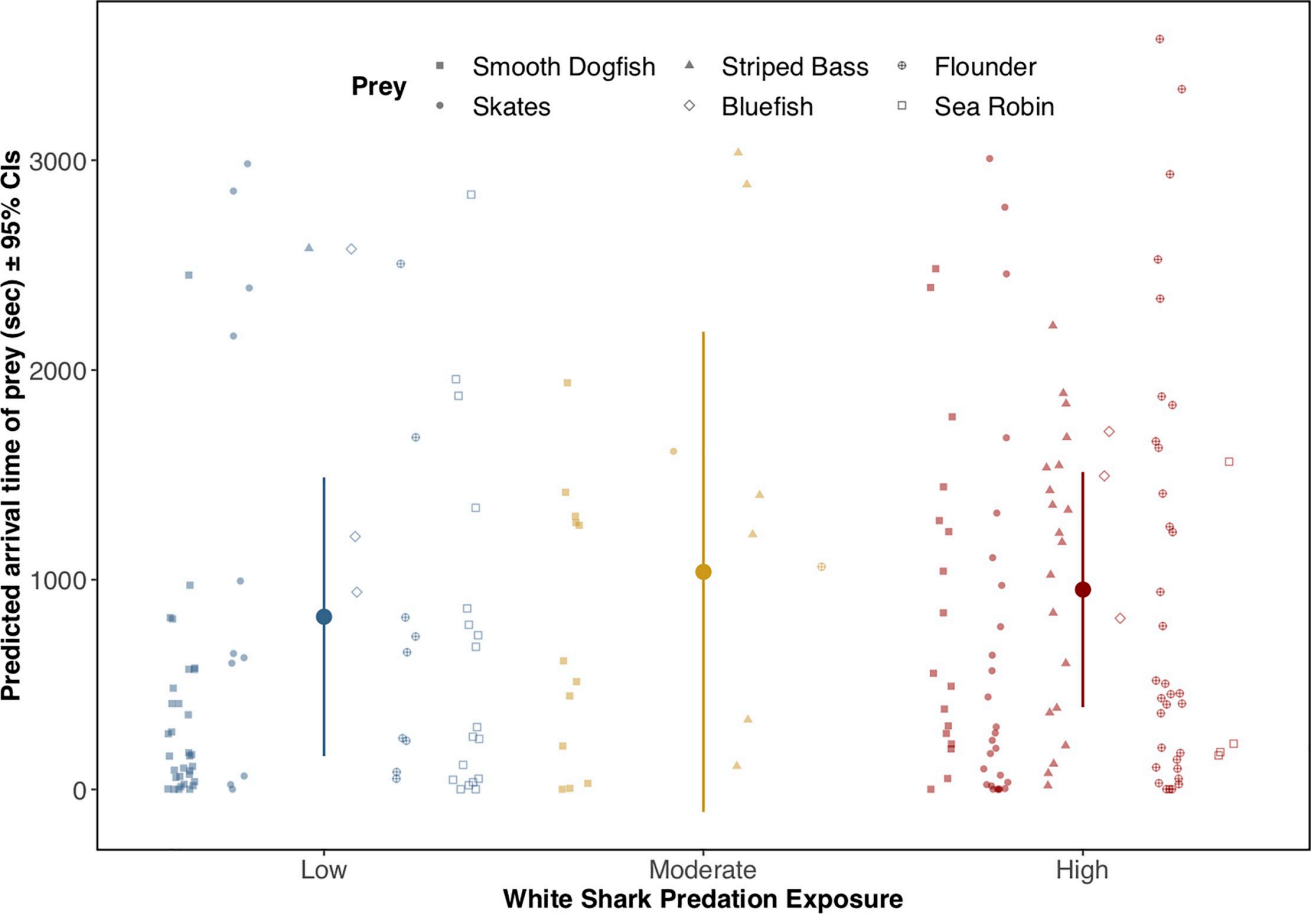
Predicted prey arrival times across a gradient of shark exposure. Predicted prey arrival times in seconds (± 95% confidence intervals) from a negative binomial GLM across varying levels of shark exposure. Predicted values are overlaid on top of raw observational data for the six prey groups.

After fitting a negative binomial GLM, a series of LRTs applied to the model showed no significant effect on overall prey arrival time resulting from shark exposure (p = 0.661; Fig 5). The interaction between prey group and exposure level, however, was significant (p<0.05; Table 2). In our model, arrival times for smooth dogfish were significantly faster (e.g., individuals took less time to arrive) at the low exposure site (negative binomial GLM; predicted arrival = 302.9 ± 10.6 seconds; p<0.01) as compared to moderate and high exposure sites (predicted MaxN = 725.8 ± 18.9 seconds and 981.5 ± 33.1 seconds, respectively), but this trend was not shared among prey groups. For example, unlike smooth dogfish, arrival times were greatest (individuals took longer to arrive) at the low exposure site for skates (p<0.01), bluefish (p<0.05), flounder (p<0.01), and sea robins (p<0.01), though neither bluefish nor sea robins occurred at the moderate exposure site.

**Table 2:** Effects of shark exposure on prey arrival times.

	Coefficients	Estimate	Standard Error	Z-value	Pr(>|z|)
	Intercept (Low + Smooth Dogfish)	7.233	0.896	8.07	<0.001*
					
**Low Exposure**	Skates	1.252	0.455	2.75	<0.01*
	Striped Bass	2.108	1.311	1.61	0.108
	Bluefish	1.575	0.781	2.02	<0.05*
	Flounder	1.450	0.451	3.21	<0.01*
	Sea Robins	1.188	0.368	3.22	<0.01*
**Moderate Exposure**	Skates	0.971	1.347	0.72	0.471
	Striped Bass	0.808	0.646	1.25	0.211
	Bluefish†	NA	NA	NA	NA
	Flounder	-0.242	1.389	-0.17	0.861
	Sea Robins†	NA	NA	NA	NA
**High Exposure**	Skates	-0.274	0.393	-0.70	0.486
	Striped Bass	0.261	0.417	0.62	0.531
	Bluefish	0.482	0.810	0.59	0.552
	Flounder	0.053	0.380	0.14	0.889
	Sea Robins	-0.550	0.713	-0.77	0.44
	Temperature	-0.082	0.046	-1.77	0.076
**Likelihood Ratio Tests (LRTs)**	**Effect**	**Likelihood Ratio ChiSq**		**Degrees of Freedom**	**Pr(>ChiSq)**
	Exposure	0.829	2	0.661
	Temperature	3.743	1	0.053
	Exposure:Prey	23.027	13	<0.05*

Summary results of a negative binomial GLM assessing the effect of shark exposure and temperature on prey arrival times and summary of LRTs.

*Indicates significance at the α = 0.05 level.

†Prey group not observed at moderate exposure site.

**Fig 6:**
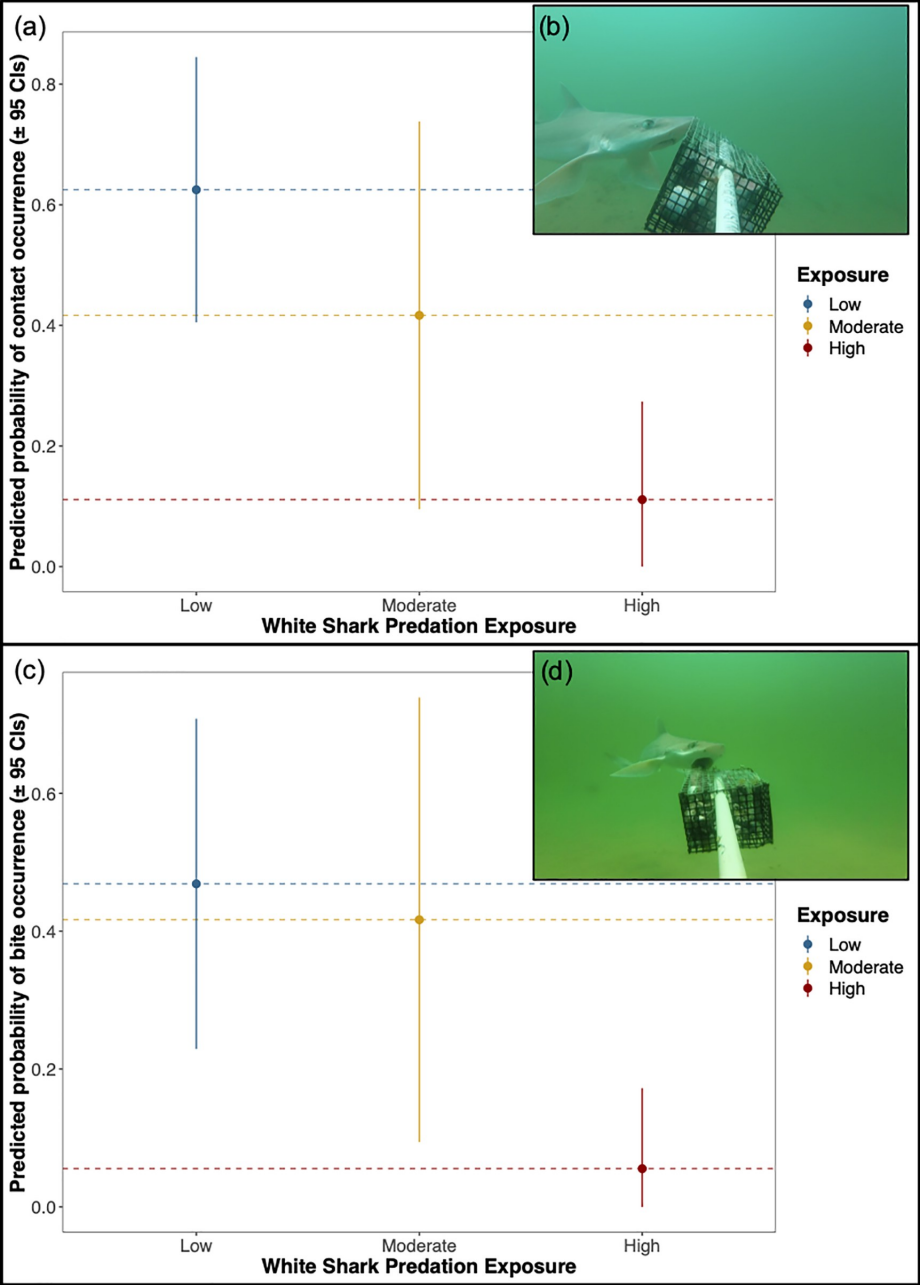
Foraging behaviors of smooth dogfish across a gradient of shark exposure. (a) Predicted occurrence (± 95% confidence intervals) of investigatory bait contact by smooth dogfish (*Mustelus canis*) at varying levels of shark exposure; (b) Still image from BRUV deployment depicting investigatory contact by smooth dogfish; (c) Predicted occurrence (± 95% confidence intervals) of bait bites by smooth dogfish; (d) Still image from BRUV deployment depicting bait bite by smooth dogfish. Sites were pooled for plotting given the range of smooth dogfish within the study area (smooth dogfish are uncommon at Race Point).

The rates of the quantified foraging behaviors (bait contacts and bites) in smooth dogfish were markedly affected by shark exposure (quasibinomial GLMs; LRTs p<0.05). Smooth dogfish were significantly less likely to make any form of bait contact and significantly less likely to make an actual bite in high exposure areas (p<0.05; Fig 6, Table 3). The predicted probabilities of bait contact or a bite occurring were reduced by factors of 5.7 and 8.4, respectively, in areas of high exposure (11.1% likelihood of contact; 5.5% likelihood of bite attempt) as compared to low exposure (62.5% likelihood of contact; 46.9% likelihood of a bite attempt). In addition to binary predictions, we performed follow-up analyses (negative binomial GLMs; see Methods) to determine whether there were differences between exposure levels (a) only where any form of bait contact, and thus, bait residency, occurred; and b) only where at least one bite attempt occurred. No trends were identified with respect to the duration of bait residency (negative binomial GLM; LRT p = 0.16) or normalized bite rates (LRT p = 0.24) when such events occurred, though temperature did influence the normalized bite rate (LRT p<0.001). We also isolated videos from moderate and high exposure sites (sites where white sharks occurred at any point in the study) to identify any potential acute effects of white shark occurrence on smooth dogfish foraging behaviors (quasibinomial GLMs; see Methods). There were no significant differences in the likelihoods of smooth dogfish contacting or biting at the bait associated with white shark occurrence in the video, suggesting a minimal influence of any acute predator avoidance behaviors on exposure-level trends (quasibinomial GLM; bait contact: LRT p = 0.06; bite: LRT p = 0.10).

**Table 3:** Effects of shark exposure on smooth dogfish foraging behaviors.

		Coefficients	Estimate	Standard Error	Z-value	Pr(>|z|)
(a)		Intercept (Low Exposure)	-4.232	2.909	-1.455	0.151
**Bait Contact**						
	Exposure	Moderate Exposure	-0.845	0.734	-1.151	0.254
		High Exposure	-2.322	0.888	-2.614	<0.05*
		Temperature	0.256	0.157	1.629	0.109
(b)		Intercept (Low Exposure)	-2.225	2.713	-0.820	0.415
**Bait Bites**						
	Exposure	Moderate Exposure	-0.185	0.706	-0.263	0.794
		High Exposure	-2.551	1.134	-2.251	<0.05*
		Temperature	0.112	0.143	0.782	0.438

Summary results of quasibinomial GLMs used to test the effects of shark exposure and temperature on smooth dogfish (*Mustelus canis*) foraging behavior, including (a) the likelihood of an individual making any form of investigatory bait contact; and, (b) the likelihood of an individual biting the bait crate.

*Indicates significance at the α = 0.05 level.

## Discussion

Predators affect their prey both via direct consumption as well as through non-consumptive, fear-based effects, which can in fact exert a stronger influence than predation itself. According to the COR hypothesis, predictable predation risk will elicit proactive, behavioral responses, while unpredictable risk will result in reactive, stress-mediated responses[34]. Behavioral responses to predictable and uncontrollable predation risk, such as alterations in habitat selection or foraging behavior, in turn may cause cascading effects throughout the ecosystem. The results of this study suggest that mobile fish species in the waters off New England may modify their behavior in response to increased exposure to, and potential predation by, large sharks such as the white shark. Overall prey abundance was significantly lower in areas of high shark exposure as compared to the low exposure site, and higher in warmer waters. Nested within this trend, however, were patterns of abundance and prey arrival times that varied amongst prey groups. In smooth dogfish, an abundant, schooling, and potentially important food item of white sharks, shark exposure resulted in lower abundances and delayed arrival times, and critically, these responses were accompanied by a modification of foraging behavior.

White sharks patrol and hunt seals along beaches where large quantities of seals haul out, however they may opportunistically prey on teleosts and elasmobranchs rather than seek them out[41]. Given this opportunistic nature of predation, many fishes, if vigilant, are likely able to avoid white shark encounters even in areas of high exposure where seal accessibility is high. In this study, we observed smooth dogfish exhibiting burst swimming away from the bait several seconds before arrival of a white shark on two separate occasions, and additionally observed a failed predation attempt by a white shark on a winter skate. However, there are energetic costs associated with increased vigilance under consistent predation risk, if uncontrolled, including declines in foraging activities or in the time an individual spends resting, which can ultimately decrease an animal’s reproductive fitness[17,53]. The presence of our bait, or any food source, within a high exposure area thus represented a food-risk trade off to fishes, though our data suggest that some groups, such as smooth dogfish and sea robins, largely avoid areas of high exposure entirely. It should be noted that the higher abundances of these species at the low exposure site may have influenced our data on dogfish foraging behaviors, as in some sharks, the propensity to pursue bait is increased in the presence of conspecifics[54].

A natural assumption regarding the non-consumptive effects of shark exposure on prey is that they should be continuous–that is to say, the effects should be strongest in areas characterized by the greatest exposure to sharks. Recent work regarding white shark predation risk off Southern Africa, however, supported the COR hypothesis that the stress response in prey is strongest when risk cannot be predicted or controlled [21], which complicates the relationship expected between risk and response. Our results support the notion that the ability of fish to mitigate potential predation risk from large sharks differs in ways that may affect whether the costs of antipredator responses are mediated by food-safety trade-offs or by stress responses. In our study, skates and flounder showed the greatest abundances at high exposure sites. This pattern likely stems from their ability to behaviorally control[34] predation risk via a refuge response, by camouflaging themselves into the sand. Other species in the study, such as smooth dogfish, lack this capacity to behaviorally mitigate predation risk. A recent study on striped bass in the same region detected no physiological stress response resulting from elevated white shark predation exposure, suggesting that, as predicted by the control of risk hypothesis [34], the costs borne by fish prey species that are exposed to predictable levels of predation risk may instead result in a food-risk trade-off[45]. The observed reduction of foraging behaviors in smooth dogfish in our study thus lends support to the control of risk hypothesis proposed in [34]. Analyzing body condition and plasma metabolites in fish prey types will help to disentangle the complexities between behavioral and physiological effects resulting from shark exposure.

The life histories, flight mechanisms, and biological characteristics of prey thus also play a role in how they respond to the threat of predation[55,56], though the response mechanisms may vary. Rather than avoiding white shark habitat or hiding, in two instances we observed adult bluefish closely following white sharks as the sharks made repeated investigatory passes by the bait, with the bluefish continuing to follow the shark (e.g., visible on each repeated pass) for more than 2 minutes in both instances. Although bluefish are common teleost prey for white sharks[43], such behavior suggests the relationship to predators is not all cost and that in some instances, bluefish may in fact not avoid white sharks at all, and instead actually pursue white sharks for the chance of scavenging scraps following a predation event.

Smooth dogfish, however, exhibited pronounced negative responses to shark exposure for all variables analyzed, including decreased abundances and a delay in mean arrival times, as well as a significant reduction in the likelihoods of bait contact or a bite attempt occurring. While we cannot discount the possible influence of abiotic factors such as the hydrodynamic regime, the effects of competition from cryptic species not detected or seen, or the potential effects of other predators such as seals, this observed decrease in smooth dogfish abundance and modification of foraging behaviors has potentially important implications. In recent decades, overfishing of groundfish (e.g., Atlantic cod, *Gadus morhua*) on the Northwest Atlantic continental shelf has been accompanied by a substantial increase of elasmobranch mesopredators, such as smooth dogfish, spiny dogfish (*Squalus acanthias*), and local species of skate[57,58]. Though the exact mechanisms by which this shift occurred are not known, it is thought that smooth dogfish and other smaller elasmobranchs may be hindering the recovery of groundfish stocks via competitive exclusion[59]. Historically, these small elasmobranchs may have been controlled due to predation by larger sharks, whose numbers had then declined[60]. Though white sharks are known to consume smooth dogfish[42–44], our study suggests that smooth dogfish may also experience nonconsumptive effects associated with predictable and consistent white shark-related predation risk, resulting in food-mediated costs with unknown consequences on dogfish condition and fitness. At this stage, the potential for these trophic interactions remain entirely speculative, however additional work on dogfish health and nutrition, as well as groundfish abundance, may help discern whether there are positive indirect impacts of white sharks on the local groundfish community.

Our experimental design resulted in several limitations which should be mentioned. Data collection was restricted to daylight hours during days of relatively calm weather. Additionally, our sampling locations comprised only a portion of the white shark habitat in the waters surrounding Cape Cod, and only included one low exposure site, located in Nantucket Sound. We cannot rule out fine-scale variations in the geological and/or hydrodynamic conditions between our low exposure site in Nantucket Sound as compared to those on the outer coast. This information was not accessible and thus could not be integrated into our models. The inclusion of additional low risk sites would lend itself to the use of mixed models in analysis, which may better account for any influence of such variation on the observed behaviors and abundances. Though we recognize the role that the hydrodynamic regime plays, in structuring local fish assemblages as well as dispersing scent plumes, our deployments were necessarily confined to at least temporarily calm waters, as rough seas or high wind and current would risk the tipping of the station. Furthermore, deployments were conducted during the summer season, when wave energy is typically low throughout the region. We cannot account for any role that potential fisheries interactions may have on the abundances and behaviors of study species. Recreational fishing is popular throughout our study area; however, actual human influence offshore, particularly around seal haul out sites, is low due to safety issues. Additional deployments in other white shark hunting areas, including within Cape Cod Bay, as well as low exposure sites further into Nantucket Sound, may provide additional context to the observed behavioral modifications. Our opportunistic analysis of occupancy to approximate exposure to prey communities helped refine our sampling design, but we recognize that our ‘moderate exposure’ site may actually function more as a ‘high exposure’ site. Long-term monitoring of shark residency will provide a more robust method for approximating potential predation risk over greater spatial and temporal scales.

Whereas detailed dietary studies on the relative contribution of mobile fish prey to the overall white shark diet are lacking[61], we provide some early evidence for potential food-web interactions with mobile fish communities, to suggest that apex predators can have impacts on prey species beyond those they may preferentially consume for high energetic gains. However, the absence of dietary data serves as a reason for careful interpretation these conclusions. This finding nonetheless highlights the ecological importance and ecosystem services of large sharks despite growing concerns related to human-wildlife conflict[24,25,62].

## Supporting information

S1 Table Statistical analyses. Summary of variables, predictors, tests and Generalized Linear Models (GLMs) used in statistical analyses.
(PDF)

S1 Fig Comparisons of temperature and depth among sites and months. Exploratory plots comparing mean depth and temperature (+/- SE) among sites and throughout the sampling periods, with overall means represented by the red horizontal lines.
(TIFF)

S1 File (XLS)
