# Supplementary material for: Effects of exposure to large sharks on the abundance and behavior of mobile prey fishes along a temperate coastal gradient
Source: PLoS One. 2020 Mar 16;15(3):e0230308. doi: 10.1371/journal.pone.0230308 (PMC7075566; doi:10.1371/journal.pone.0230308)
Supplement: S1 Table — Summary of variables, predictors, tests and Generalized Linear Models (GLMs) used in statistical analyses. (PDF) [file pone.0230308.s001.pdf]

Predator/Prey Group	Response Variable(s)	Predictor(s)	Model / Test
White Sharks	White Shark Occupancy	Site	Welch's T-Tests
All Prey Groups	Prey Abundance (MaxN)	Shark Exposure<br>Temperature<br>Exposure:Prey Group	Negative Binomial GLM
	Prey Arrival Time		Negative Binomial GLM
Smooth Dogfish	Bait Contact (Y/N)	Shark Exposure<br>Temperature	Quasibinomial GLM
	Bait Residency		Negative binomial GLM
	Bite Occurrence (Y/N)		Quasibinomial GLM
	Normalized Bite Count		Negative binomial GLM
Smooth Dogfish<br>(high/moderate exposure)	Bait Contact (Y/N)	White Shark Occurrence (Y/N)<br>Temperature	Quasibinomial GLM
	Bite Occurrence (Y/N)		Quasibinomial GLM
