# Supplementary figures and images for: Effects of exposure to large sharks on the abundance and behavior of mobile prey fishes along a temperate coastal gradient
Source: PLoS One. 2020 Mar 16;15(3):e0230308. doi: 10.1371/journal.pone.0230308 (PMC7075566; doi:10.1371/journal.pone.0230308)

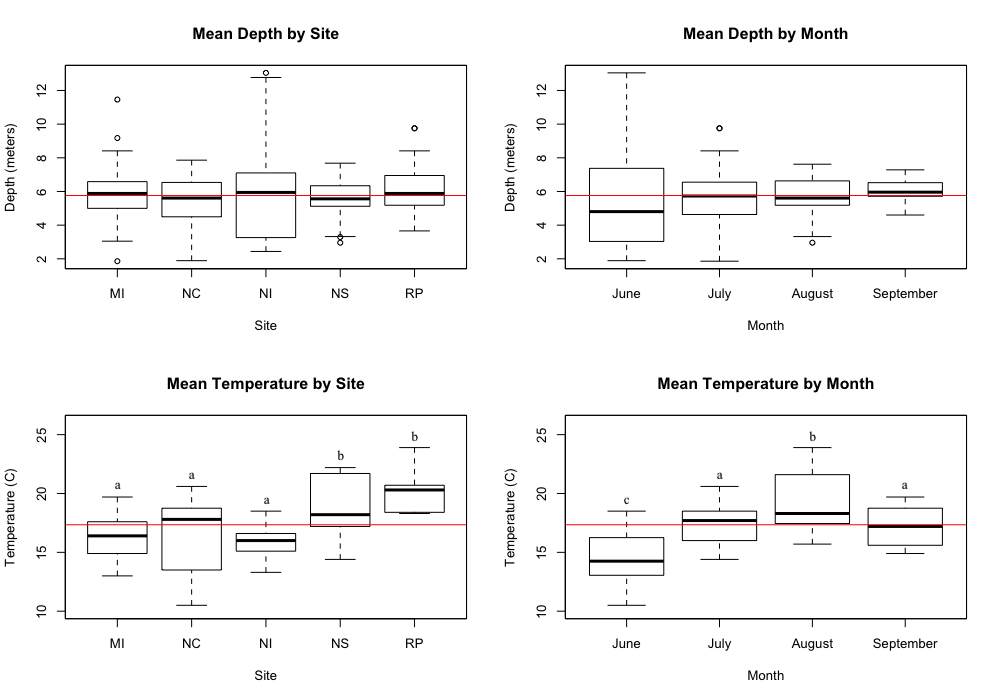

Supplement: S1 Fig — Exploratory plots comparing mean depth and temperature (+/- SE) among sites and throughout the sampling periods, with overall means represented by the red horizontal lines. (TIFF) [file pone.0230308.s002.tiff]
